# Predicting the surgical difficulty of laparoscopic cholecystectomy after percutaneous transhepatic gallbladder drainage in patients with acute cholecystitis: a multi-center study

**DOI:** 10.1007/s13304-026-02526-9

**Published:** 2026-02-06

**Authors:** Nan Yang, Wei Zhang, Jiaming Liu, Cheng Yang, Zhenghua Ding

**Affiliations:** 1 Department of General Surgery, Xiangyang No 1 People’s Hospital, Hubei University of Medicine, 441000 Xiangyang City, Hubei Province China; 2 The First Affiliated Hospital of Jinzhou Medical University, 121000 Jinzhou City, Liaoning Province China; 3 Jinzhou Central Hospital, 121000 Jinzhou City, Liaoning Province China

**Keywords:** Percutaneous transhepatic gallbladder drainage, Laparoscopic cholecystectomy, Acute cholecystitis, Machine learning, Risk factors, Surgical difficulty

## Abstract

This study aimed to establish a predictive model to evaluate the surgical difficulty of laparoscopic cholecystectomy (LC) after percutaneous transhepatic gallbladder drainage (PTGBD) in patients with acute cholecystitis (AC) using preoperative parameters. Clinical data from 580 patients who underwent LCs at three tertiary hospitals between January 2013 and June 2024 were retrospectively analyzed. Predictors were selected through LASSO and Logistic analyses. Eight machine learning algorithms were used to create prediction models, evaluated by ten-fold cross-validation, ROC curves, DCA, calibration curves, precision-recall curves, confusion matrices, and DeLong’s test. SHAP analysis enhanced model interpretability. The intervals between onset and PTGBD, upper abdominal surgery histories, WBC, gallstones, gallbladder wall thickness, neutrophil percentage (Neut%), and time until PTGBD were identified as predictors. The Naive Bayes (NB) model demonstrated outperformed other models in clinical decision-making and prediction. SHAP analysis identified Neut% as the most critical prediction model feature, followed by the time until surgery after PTGBD. A online calculator was developed using the NB model (https://zw17786325639.shinyapps.io/difficulty/). This study developed and verified a network calculator based on the NB model to predict LC surgical difficulty after PTGBD, aiding surgeons in preoperative risk assessment and timing selection.

## Introduction

Acute cholecystitis (AC) caused by gallstones is a common acute abdominal condition encountered in clinical practice, and laparoscopic cholecystectomy (LC) remains the gold standard for surgical treatment [1]. However, in patients with high-risk AC and severe underlying diseases or unstable general conditions (such as older adult patients or those with impaired cardiopulmonary function), the risk during emergency LC surgery is significantly high [2]. For such patients, the Tokyo Guidelines (2018) (TG18) recommend a staged treatment strategy, starting with percutaneous transhepatic gallbladder drainage (PTGBD) to control infection and inflammation, followed by LC after the condition has stabilized, thereby reducing surgical risk [2, 3]. This approach is beneficial because it can reduce local gallbladder edema and improve systemic symptoms, thus creating better conditions for surgery [4, 5]. However, several factors can lead to severe adhesions around the gallbladder during LC after PTGBD, making surgical procedures more difficult, necessitating conversion to open surgery, and potentially causing serious and life-threatening complications [6]. Individual differences exist regarding surgical difficulty, and an accurate assessment of surgical difficulty is of great significance in reducing surgical risks, improving surgical success rates, and minimizing unnecessary waste of medical resources [7]. Traditional methods for assessing surgical difficulty mainly rely on the clinician’s experience and subjective judgment, which lack objectivity and accuracy. The integration of artificial intelligence and medicine is increasing, and machine learning algorithms can process complex data and mine potential patterns and rules, thereby providing new methods and ideas for surgical difficulty assessment.

The purpose of our research was to develop a web-based calculator using the best machine learning model to predict the surgical difficulty of LC in patients with AC after PTGBD, formulate personalized medical strategies, and avoid intraoperative complications to achieve better postoperative outcomes.

## Methods

### Patients

This study was approved by the Institutional Research and Ethics Committee of Jinzhou Central Hospital (Ethics Number: JZZXYYLL20250103-2). All procedures involving human participants were conducted in accordance with the Declaration of Helsinki (Revised 2013).

A multicenter retrospective analysis was conducted on 812 patients with AC who were admitted to Jinzhou Central Hospital, First Affiliated Hospital of Jinzhou Medical University, and Xiangyang No 1 People’s Hospital from January 2013 to June 2024. The strict adherence to the inclusion and exclusion criteria resulted in a final inclusion of 580 patients.

The inclusion criteria were as follows: (1) Patients who underwent percutaneous transhepatic gallbladder puncture and catheterization combined with LC, including those who required conversion to open cholecystectomies. (2) The patient was diagnosed with AC according to the TG18 criteria [8, 9] at the time of admission, and histopathological examination results after LC confirmed AC. (3) The patient had complete and available clinical data and related information from both admissions.

The exclusion criteria were as follows: (1) Histopathological results showing malignancy. (2) Patients who did not undergo LC because of complex conditions or other serious diseases. (3) Patients with severe complications such as gallbladder leakage after surgery and death. (4) Patients with missing clinical or incomplete data related to the study.

Difficult LC is defined as the presence of one of the following: switching from LC to open cholecystectomy with a procedure time of ≥ 120 min, or blood loss of ≥ 300 mL [5, 6, 10]. The conversion criteria were as follows: other organ damage, refractory hemorrhage, and difficulty in identifying vital structures within the Calot triangle [11–13]. Patients were divided into two groups according to the definition of difficult LC: the difficult LC (DLC) group (275 patients) and the non-difficult LC (NDLC) group (305 patients). Depending on the team of physicians performing the LCs, patients from Jinzhou Central Hospital were used as the training dataset (406 patients), and patients from the remaining two hospitals were used as the validation dataset (174 patients).

The three institutions involved in this study were tertiary hospitals qualified for biliary surgery. The indications for PTGBD uniformly followed the TG18 standards, namely, “moderate/severe cholecystitis unable to tolerate emergency surgery, severe underlying diseases (such as heart failure, liver failure), and high risk of gallbladder necrosis or perforation.” The procedures were performed by physicians with more than 5 years of experience in interventional ultrasound. Indications for surgery were mild cholecystitis and moderate/severe cholecystitis that stabilized after PTGBD treatment. Patients with biliary obstruction due to common bile duct stones, gallbladder cancer, or severe coagulation dysfunction were excluded. All surgeons participated in unified technical training. The surgeries were primarily performed by experienced surgical teams (all with the title of associate chief physician or higher, having performed more than 500 LCs, and more than 100 other laparoscopic surgeries), and there was no significant difference in the composition of the surgeons across the institutions. Intraoperative cholangiography (IOC) was performed by the surgeon based on intraoperative findings or preoperative suspicion of common bile duct stones.

### Research variables

Data were extracted from the electronic medical records of each patient and included the following variables: demographic information (sex and age), interval between onset and PTGBD (defined as the time from symptom presentation to PTGBD), and time until surgery after PTGBD (defined as the interval from PTGBD to LC). Clinical presentations included a history of smoking, alcohol consumption, positive Murphy’s sign, a cholecystitis history, and temperature. Abdominal surgical history included upper abdominal surgery, lower abdominal surgery, laparoscopic surgery, and laparotomy. Chronic disease history included diabetes mellitus, hypertension, coronary heart disease, lung disease, and cerebrovascular disease. Abdominal ultrasonography findings included gallstones, gallbladder wall thickness (GBWT), gallbladder diameter (GBD), and pericholecystic effusions. Laboratory test results included white blood cell (WBC) count level, hemoglobin (HB), prothrombin time (PT), international normalized ratio (INR), total bilirubin (TBIL) level, aspartate aminotransferase (AST) level, alanine aminotransferase (ALT) level, gamma-glutamyl transferase (GGT) level, alkaline phosphatase (ALP) level, blood urea nitrogen (BUN) level, serum creatinine (SCR) level, neutrophil percentage (Neut%), platelet (PLT) count, lymphocyte (LYM) count, C-reactive protein (CRP) level, and platelet-to-lymphocyte ratio (PLR).

### Feature selection

Clinical features were subjected to statistical preprocessing and analysis. Considering that imputation methods for handling high levels of missing data may introduce additional assumptions that could affect model interpretability, we excluded these features during the preprocessing phase to ensure the credibility of our model. During preprocessing, we standardized all numerical features to ensure comparability across scales. Given the large number of clinical features in this study and the potential for multicollinearity, feature selection was crucial for identifying optimal predictive features and preventing overfitting. To mitigate potential overfitting, we initially employed the LASSO regression analysis to select the most representative features. The optimal regularization parameter (λ) was determined through tenfold cross-validation, and features with nonzero coefficients were selected as candidates. Subsequently, multivariate logistic regression analysis was conducted on these candidate features, and variables with *P*-values < 0.05 were selected as final predictive features. Additionally, Pearson’s correlation analysis was used to calculate the correlation coefficients between pairs of predictive features, and pairs with high redundancy (|r| > 0.9) were manually excluded to further reduce multicollinearity. The remaining features were retained for downstream modeling.

### Machine learning algorithms

We selected eight widely used classifier algorithms: Logistic Regression (LR), Decision Tree (DT), Random Forest (RF), Extreme Gradient Boosting (XGB), Support Vector Machine (SVM), Multilayer Perceptron (MLP), K-Nearest Neighbor (KNN), and Naive Bayes (NB). For the tree-based models (DT, RF, and XGB), feature importance scores were calculated based on the mean decrease in the Gini impurity. All machine learning models were trained using R software (v 4.4.1). The training dataset utilized ten-fold cross-validation, whereas the validation dataset was used directly for model validation. To evaluate the model performance and test clinical applicability, we used the area under the receiver operating characteristic (ROC) curve (AUROC), calibration curves, decision curve analysis (DCA), precision-recall (PR) curves, and confusion matrices. Delong’s test was used to assess the differences in AUROC values among models. Shapley Additive Interpretation (SHAP) analysis was employed to rank feature importance and provide personalized model explanations. Based on a model with optimal performance, we developed a web-based calculator to assess the difficulty of LC following PTGBD.

### Statistical analyses

Statistical analyses were conducted using the R software (version 4.4.1). The Kolmogorov–Smirnov test was used to assess the normality of continuous variables. Features with normal distribution were compared using Student’s t-test and presented as means ± standard deviations (SDs). Non-normally distributed features were analyzed using the Kruskal–Wallis rank-sum test and presented as medians and interquartile ranges (IQRs). Categorical variables were compared using the chi-squared test (χ²) and presented as counts and percentages (%). Statistical significance was defined as a two-sided *P*-value of < 0.05.

## Results

### Baseline characteristics

This was a retrospective analysis of 812 patients with AC. After applying the exclusion criteria, 580 patients were included in the final analysis. According to the definition of difficult LC, patients in the training set were divided into two groups: the NDLC group (*n* = 211, 51.97%) and the DLC group (*n* = 195, 48.03%), comprising 217 males and 189 females. Table 1 shows the baseline patient characteristics. Significant differences were observed between the NDLC and DLC groups in terms of the interval between disease onset and PTGBD, history of upper abdominal surgery, history of laparotomy, WBC count, gallstones, GBWT, Neut%, and time until surgery after PTGBD (all *P* < 0.05). Specifically, compared with the NDLC group, the DLC group had a higher prevalence of a history of upper abdominal surgery (52.8% vs. 30.8%) and laparotomies (31.8% vs. 20.4%). Laboratory markers were significantly elevated in the DLC group, including WBC counts (15.44 [8.6] vs. 11.5 [8.605]) and Neut% (11.5 [9.21] vs. 6.72 [5.475]). Ultrasonographic parameters also differed significantly between the groups, with the DLC group showing higher rates of gallstones (61.5% vs. 41.7%) and higher GBWTs (0.45 [0.385] vs. 0.37 [0.25]). The interval between disease onset and PTGBD was 96 (84) hours in the NDLC group and 144 (96) hours in the DLC group. The time until surgery after PTGBD was 38 (16) days in the NDLC group and 54 (28) days in the DLC group. Through randomization with a balanced data distribution, no statistically significant differences were found between the training and validation sets in terms of the variables (*P* > 0.05).

**Table 1:** Comparison of baseline characteristics of patients

Characteristic	NDLC group (*N* = 211 )	DLC group (*N* = 195)	*P*-value	Training set (*N* = 406)	Validation set (*N* = 174)	*P*-value
Sex, n (%)			0.5875			0.8701
Male	116 (55.0%)	101 (51.8%)		217 (53.45%)	95 (54.60%)	
Female	95 (45.0%)	94 (48.2%)		189 (46.55%)	79 (45.40%)	
Age (years)	72.41 (5.93)	72.98 (5.86)	0.3332	72.68 (5.89)	72.74 (6.67)	0.9228
Interval between onset and PTGBD (h)	96 (84)	144 (96)	**< 0.001** ^*^	96 (96)	120 (96)	0.334
History of smoking, n (%)	65 (30.81%)	59 (30.26%)	0.9903	124 (30.5%)	41 (23.6%)	0.1081
History of alcohol consumption, n (%)	93 (44.08%)	87 (44.62%)	0.9925	180 (44.3%)	71 (40.8%)	0.4871
Murphy’s sign positive, n (%)	165 (78.20%)	150 (76.92%)	0.8502	315 (77.6%)	143 (82.2%)	0.2569
History of cholecystitis, n (%)	50 (23.7%)	55 (28.2%)	0.356	105 (25.86%)	48 (27.59%)	0.7422
History of upper abdominal surgery, n (%)	65 (30.8%)	103 (52.8%)	**< 0.001** ^*^	168 (41.38%)	75 (43.10%)	0.8686
History of lower abdominal surgery, n (%)	30 (14.2%)	15(7.7%)	0.0531	45 (11.08%)	23 (13.22%)	0.5542
History of laparoscopic surgery, n (%)	46 (21.8%)	57 (29.2%)	0.1085	45 (11.08%)	23 (13.22%)	0.3059
History of laparotomy, n (%)	43 (20.4%)	62 (31.8%)	**0.012** ^*^	105 (25.86%)	43 (24.71%)	0.8516
Diabetes mellitus, n (%)	61 (28.9%)	63 (32.3%)	0.5256	124 (30.542%)	53 (30.460%)	> 0.99
Hypertension, n (%)	70 (33.2%)	73 (37.4%)	0.4272	143 (35.2%)	71 (40.8%)	0.2368
Coronary heart disease, n (%)	55 (26.066%)	51 (26.154%)	> 0.99	106 (26.1%)	55 (31.6%)	0.2097
Lung disease, n (%)	36 (17.06%)	31 (15.90%)	0.8556	67 (16.50%)	23 (13.22%)	0.3811
Cerebrovascular disease, n (%)	34 (16.1%)	22 (11.3%)	0.2053	56 (13.79%)	23 (13.22%)	0.9579
Temperature (℃)	37.88 (0.86)	37.81 (0.83)	0.3754	37.8 (1.3)	37.9 (1.2)	0.572
WBC (× 10^9^/L)	11.5 (8.605)	15.44 (8.6)	**< 0.001** ^*^	13.25 (9.095)	14.04 (6.7625)	0.0765
Gallstones, n (%)	88 (41.7%)	120 (61.5%)	**< 0.001** ^*^	208 (51.2%)	95 (54.6%)	0.5137
GBWT (mm)	0.37 (0.25)	0.45 (0.385)	**< 0.001** ^*^	0.4 (0.34)	0.36 (0.45)	0.17
GBD (cm)	7.64 (2.19)	7.42 (1.66)	0.254	7.3 (2.73)	6.95 (1.43)	0.466
Pericholecystic effusion	154 (73.0%)	131 (67.2%)	0.2423	285 (70.2%)	110 (63.2%)	0.1199
HB (g/L)	145 (16)	147 (14.5)	0.0602	146 (14)	145 (16.75)	0.465
PT (s)	13.5 (2.45)	13.5 (2.5)	0.727	13.5 (2.4)	13.5 (2.2)	0.42
INR	1.25 (0.205)	1.27 (0.185)	0.557	1.26 (0.19)	1.27 (0.21)	0.783
TBIL (µmol/L)	26.6 (47.24)	28.3 (50.25)	0.594	27.3 (48.5)	26.1 (18.3)	0.527
AST (U/L)	41 (35)	37 (27)	0.439	40 (30)	39 (44.75)	0.223
ALT (U/L)	44 (38.5)	45 (33.5)	0.926	44.5 (36)	46.5 (34)	0.292
GGT (U/L)	57 (39.5)	59 (41)	0.704	57.5 (40.75)	59 (43.75)	0.154
ALP (U/L)	96 (60.5)	100 (45.5)	0.241	98 (52)	102.5 (73)	0.707
BUN mmol/L	7.7 (2.4)	7.2 (2.55)	0.103	7.5 (2.4975)	7.3 (2.2)	0.809
SCR µmol/L	84 (25.12)	80.32 (21.41)	0.1113	84.25 (36)	86.4 (25.325)	0.132
Neu (×10^9^/L)	6.72 (5.475)	11.5 (9.21)	**< 0.001** ^*^	9.09 (7.5975)	8.76 (10.7925)	0.238
PLT (×10^9^/L)	202.1(55.65)	201.3(49.2)	0.611	201.4(52.25)	201.5 (40.3)	0.813
LYM (×10^9^/L)	0.85 (0.475)	0.82 (0.48)	0.343	0.84 (0.49)	0.86 (0.54)	0.783
CRP (mg/L)	7.5 (15.95)	7.1 (12.95)	0.95	7.25 (14.85)	8.4 (14.85)	0.599
PLR	232 (144.627)	238 (140.5)	0.844	235 (144.582)	229.5 (131.993)	0.875
Time until surgery after PTGBD (days)	38 (16)	54 (28)	**< 0.001** ^*^	42 (25.75)	40 (30.5)	0.202

Bold highlights statistically significant differences (*P*<0.05)

* represents *P* < 0.05

NDLC, non-difficult laparoscopic cholecystectomy; DLC, difficult laparoscopic cholecystectomy; PTGBD, percutaneous transhepatic gallbladder drainage; WBC, white blood cell count; GBWT, gallbladder wall thickness; GBD, gallbladder diameter; HB, hemoglobin; PT, prothrombin time; INR, international normalized ratio; TBIL, total bilirubin; AST, aspartate aminotransferase; ALT, alanine aminotransferase; GGT, gamma-glutamyl transferase; ALP, alkaline phosphatase; BUN, blood urea nitrogen; SCR, serum creatinine; Neu%, neutrophil percentage; PLT, platelet count; LYM, lymphocyte count; CRP, C-reactive protein; PLR, platelet-to-lymphocyte ratio

### LASSO and logistic regression for model development feature selection

**Fig. 1:**
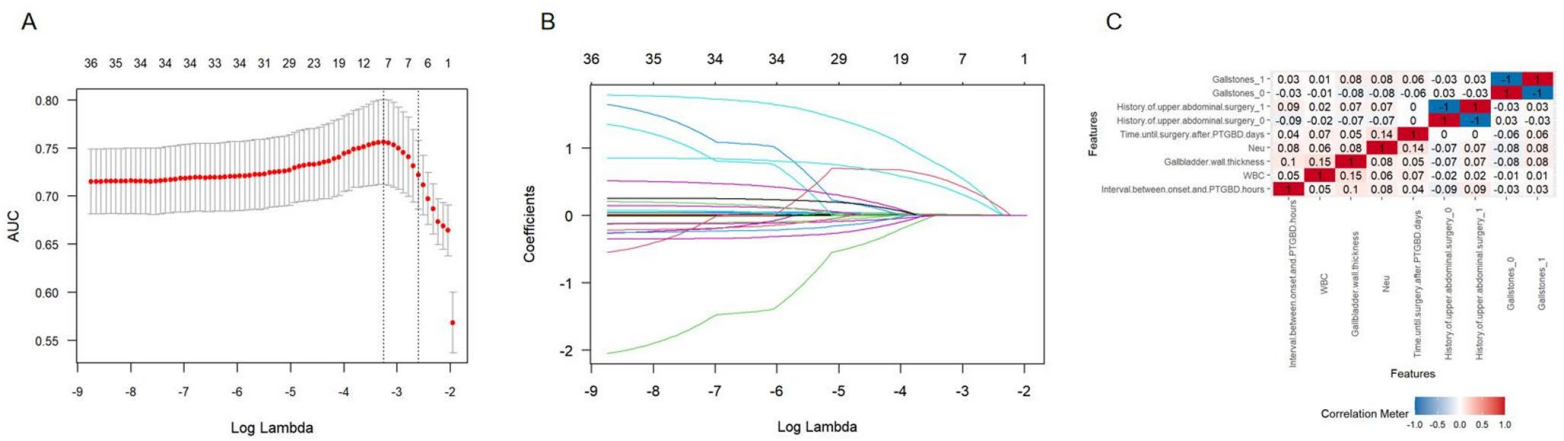
Feature selection results. **A**, **B** Clinical feature selection based on the LASSO regression model. **C** The correlation heatmap between the independent clinical variables. AUC, area under the curve; PTGBD, percutaneous transhepatic gallbladder drainage; Neu, neutrophil percentage; WBC, white blood cell

For feature selection in model development, LASSO regression analysis was employed, resulting in the identification of seven features with nonzero coefficients (Fig. 1A–B). Subsequently, multivariate logistic regression analysis was conducted on these features to adjust for potential confounding factors, ultimately determining seven independent risk factors: interval between onset and PTGBD (odds ratio [OR] = 1.01, 95% confidence interval [CI]: 1.00–1.01, *P* < 0.001), history of upper abdominal surgery (OR = 2.52, 95% CI: 1.59–4.03, *P* < 0.001), WBC count (OR = 1.05, 95% CI: 1.01–1.09, *P* = 0.025), gallstones (OR = 2.24, 95% CI: 1.42–3.56, *P* < 0.001), GBWT (OR = 3.83, 95% CI: 1.39–11.00, *P* = 0.011), Neut% (OR = 1.04, 95% CI: 1.01–1.07, *P* = 0.003), and time until surgery after PTGBD (OR = 1.03, 95% CI: 1.02–1.05, *P* < 0.001) (Table 2). Additionally, Pearson’s correlation analysis was performed on these features, and the correlation heatmap demonstrated their independence, indicating the absence of multicollinearity (Fig. 1C). Machine-learning models were established based on these seven independent predictors.

**Table 2:** Multivariate logistics regression analysis

Variable	OR	95% CI	*P*-value
Interval between onset and PTGBD (hours)	1.01	1.00–1.01	< 0.001*
History of upper abdominal surgery	2.52	1.59–4.03	< 0.001*
WBC	1.05	1.01–1.09	0.025
Gallstones	2.24	1.42–3.56	< 0.001*
GBWT	3.83	1.39–11.00	0.011
Neu	1.04	1.01–1.07	0.003
Time until surgery after PTGBD (days)	1.03	1.02–1.05	< 0.001*

*represents *P* < 0.001

OR, odds ratio; 95% CI, 95% confidence interval; WBC, white blood cell; GBWT, gallbladder wall thickness; Neu, neutrophil percentage; PTGBD, percutaneous transhepatic gallbladder drainage

### Performance of developed models

**Fig. 2:**
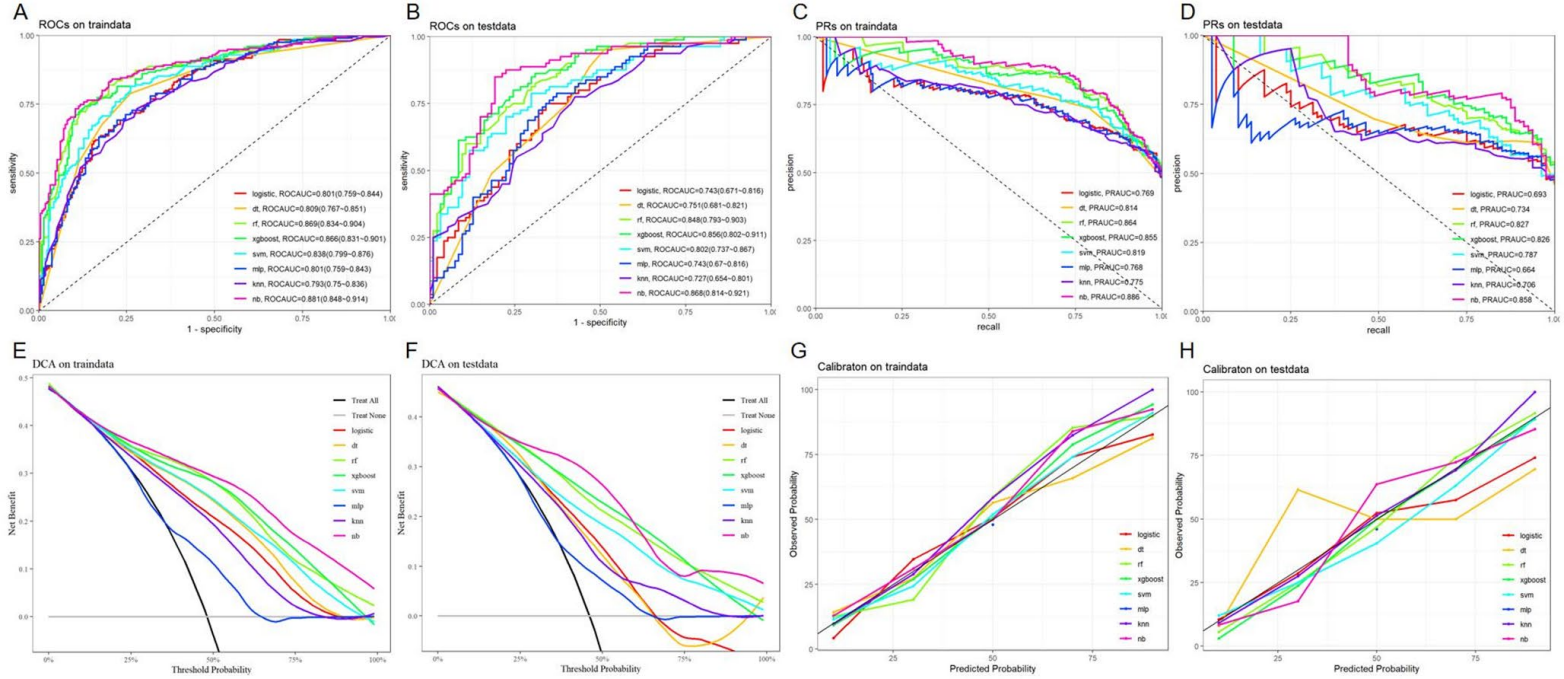
Construction and performance evaluation of eight machine learning models. **A, B** Receiver operating characteristics (ROC) curves of eight machine learning models in the training set **(A)** and validation set **(B)**. **C, D** The precision-recall (PR) curves of eight machine learning models in the training set **(C)** and validation set **(D)**. **E, F** The decision curve analysis of eight machine learning models in the training set **(E)** and validation set **(F)**. **G, H** Calibration curves of eight machine learning models in the training set **(G)** and validation set **(H)**. LR, logistic regression; DT, decision tree; RF, random forest; XGB, Extreme Gradient Boosting; SVM, support vector machine; MLP, multilayer perceptron; KNN, K-Nearest Neighbor; NB, naive bayes

**Fig. 3:**
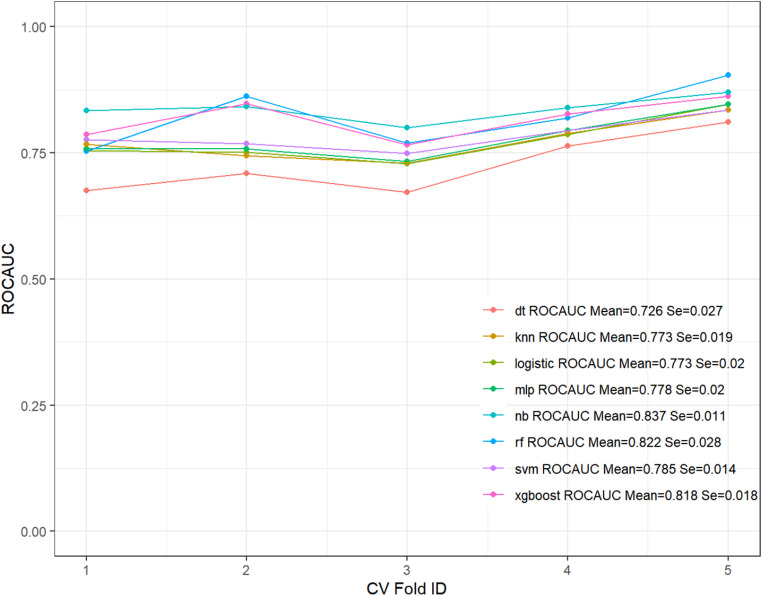
Ten-fold cross-validation results of eight machine learning models in the training set

**Fig. 4:**
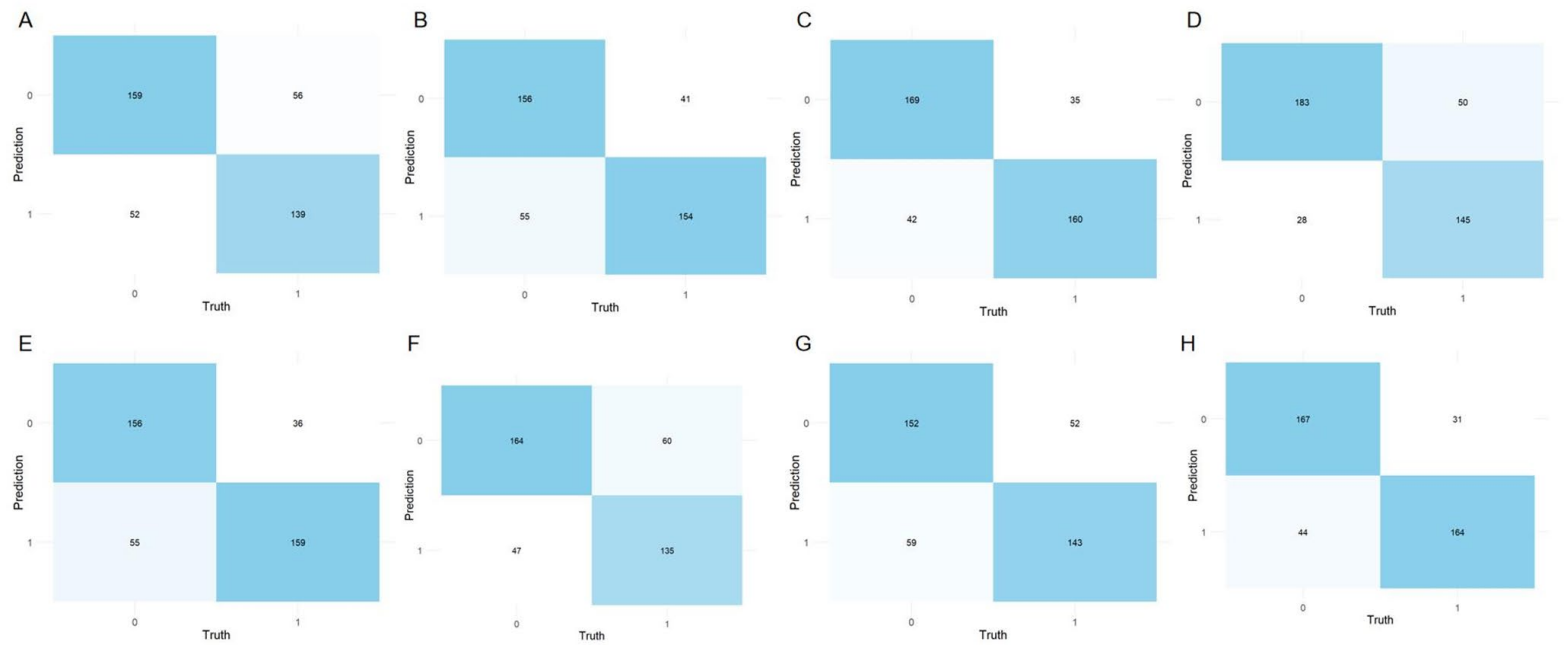
Confusion matrices for eight machine learning models in the training set. **A** Logistic regression. **B** Decision tree. **C** Random forest. **D** Extreme Gradient Boosting. **E** Support vector machine. **F** Multilayer perceptron. **G** K-Nearest Neighbor. **H** Naive bayes

**Fig. 5:**
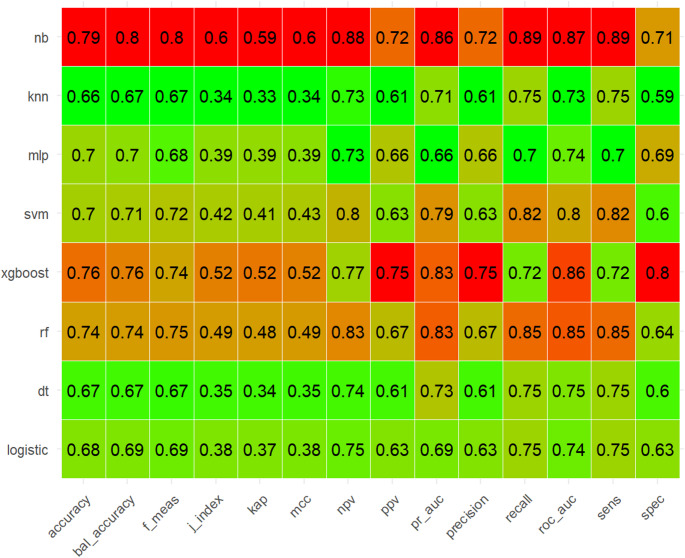
Prediction performance of eight machine learning models in the training set. nb, naive bayes; knn, K-Nearest Neighbor; mlp, multilayer perceptron; svm, support vector machine; xgboost, extreme gradient boosting; rf, random forest; dt, decision tree; logistic, logistic regression

In the training set, the NB model showed superior prediction performance compared to the other seven models, with an AUROC value of 0.881 (95% CI: 0.848–0.914) (Fig. 2A). In the validation set, the NB model also outperformed the other models, with an AUROC of 0.868 (95% CI: 0.810–0.921) (Fig. 2B). PR curves were generated to complement the ROC curves and further assess the strengths and weaknesses of the models. Figures 2C–D show that the NB model had a higher average area under the precision–recall (AUPRC) than the other models (training set AUPRC, 0.886; validation set AUPRC, 0.858). In the DCA and calibration curves of both the training and validation sets, the NB model exhibited good clinical decision-making and actual prediction abilities compared to the other seven models (Fig. 2E–H). Furthermore, the performance of the eight machine learning algorithms was evaluated using ten-fold cross-validation, with the NB model showing the best performance, with an average AUROC value of 0.837 (Fig. 3). The confusion matrices of the different models demonstrated that the NB model performed better in detecting both positive and negative samples, showing a higher sensitivity (Fig. 4). Compared with the other models, the NB model had higher accuracy, positive predictive value (PPV), precision, recall, and sensitivity (Fig. 5). DeLong’s test showed no significant differences in the AUROC among the eight models (*P* > 0.05), indicating no overfitting of the models (Table 3).

**Table 3:** Different machine learning models’ performance

Machine learning models	AUC	95% CI	*P*-value
LR	0.801	0.759–0.844	0.1768
DT	0.809	0.767–0.851	0.1615
RF	0.869	0.834–0.904	0.5246
XGB	0.866	0.831–0.901	0.7673
SVM	0.838	0.799–0.876	0.3553
MLP	0.801	0.759–0.843	0.1787
KNN	0.793	0.750–0.836	0.1342
NB	0.881	0.848–0.914	0.6898

Abbreviations: AUC, area under the curve; LR, logistic regression; DT, decision tree; RF, random forest; XGB, Extreme Gradient Boosting; SVM, support vector machine; MLP, multilayer perceptron; KNN, K-Nearest Neighbor; NB, naïve bayes

### Variable importance

**Fig. 6:**
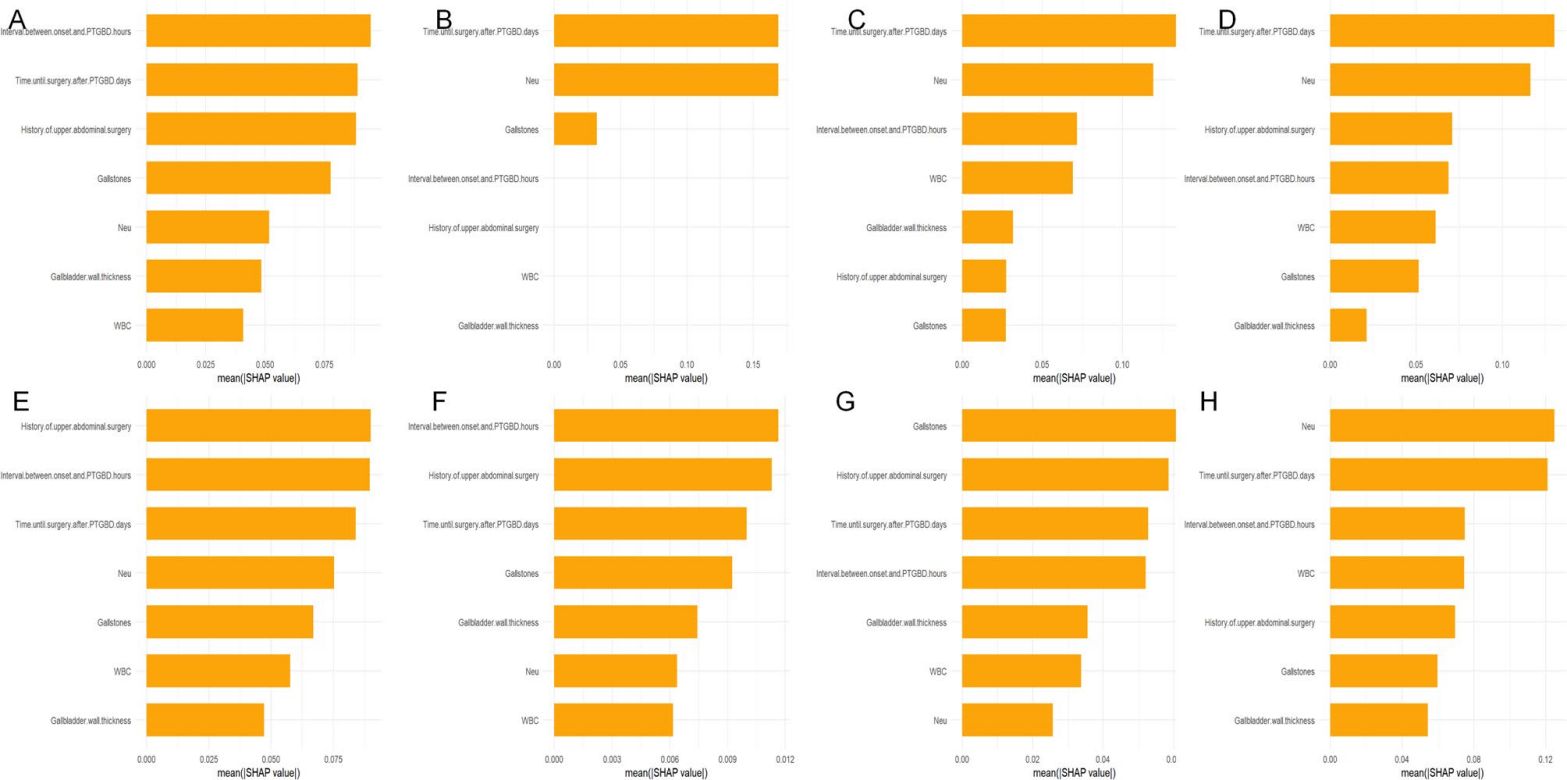
Importance ranking plot of independent clinical variables of eight machine learning models. **A** Logistic regression. **B** Decision tree. **C** Random forest. **D** Extreme Gradient Boosting. **E** Support vector machine. **F** Multilayer perceptron. **G** K-Nearest Neighbor. **H** Naïve bayes

SHAP analysis was employed to assess the importance of the clinical features and individual prediction contributions within each machine learning algorithm (Fig. 6). The results indicated that the feature “Time until surgery after PTGBD” had relatively high predictive importance across all machine learning algorithms. In the NB model, the importance ranking of the variables was as follows: Neut%, time until surgery after PTGBD, interval between onset and PTGBD, WBC count, history of upper abdominal surgery, gallstones, and GBWT (Fig. 6H). Among them, Neut% had the highest predictive importance, followed by the time until surgery after PTGBD. The higher the ranking of a variable, the greater its contribution to predicting the difficulty of LC surgery after PTGBD in patients with AC. Therefore, in clinical practice, we should promptly and cautiously monitor and deal with these related indicators.

### Model explainability

**Fig. 7:**
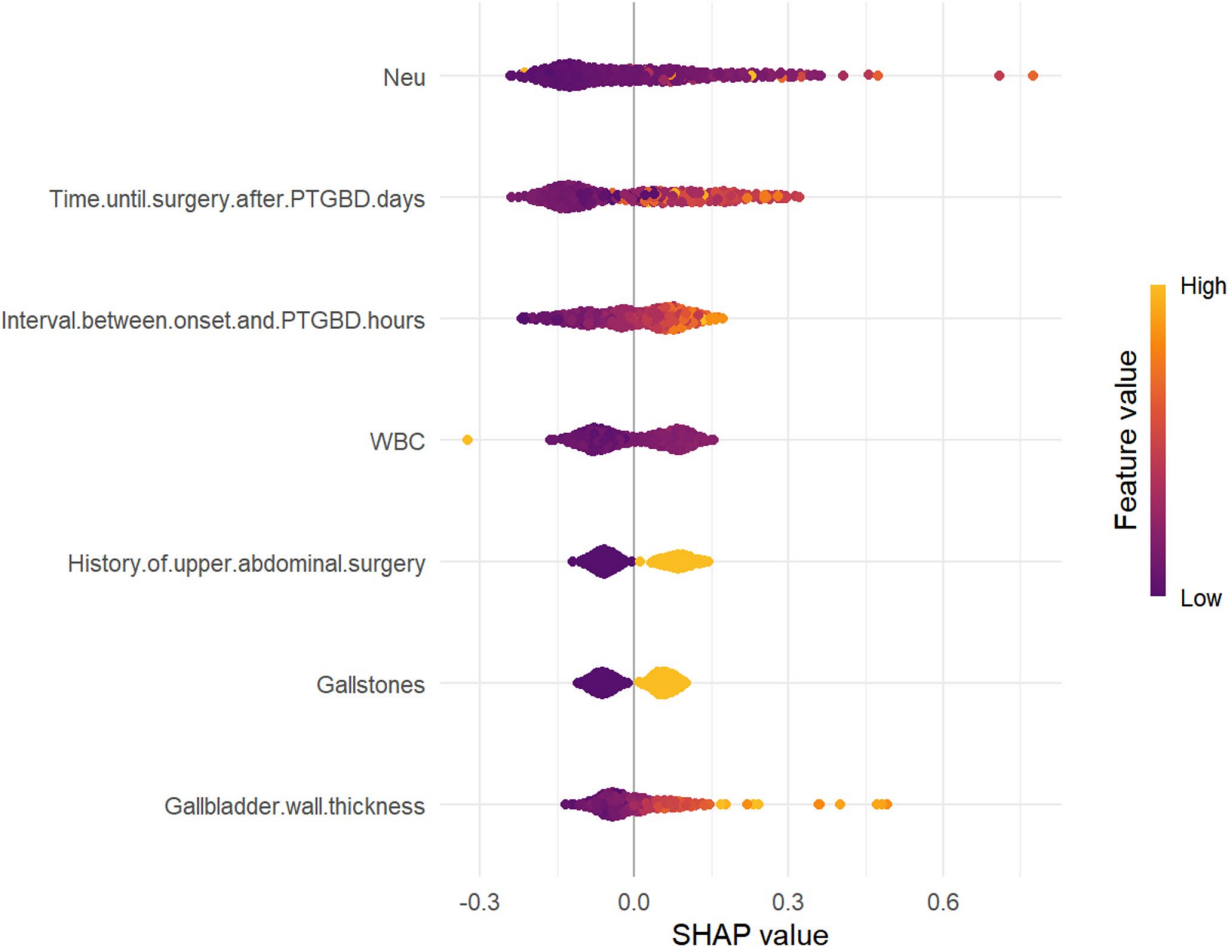
SHAP summary chart of independent clinical variables in the naive bayes model. Neu, neutrophil percentage; PTGBD, percutaneous transhepatic gallbladder drainage; WBC, white blood cell

**Fig. 8:**
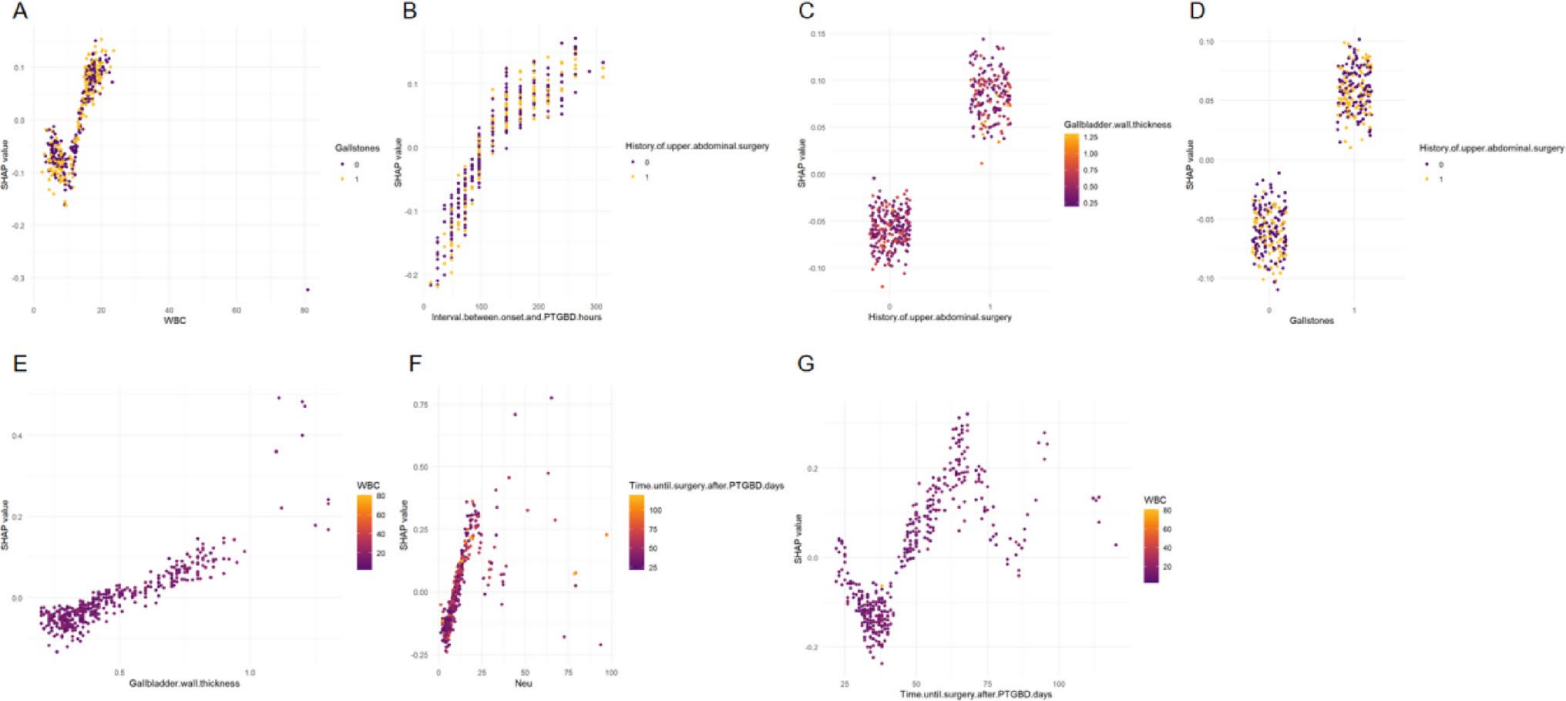
SHAP dependency plots of the seven influential clinical features on model outcome. Neu, neutrophil percentage; PTGBD, percutaneous transhepatic gallbladder drainage; WBC, white blood cell

To further improve the clinical utility of the model, the SHAP method was used to determine clinical indicators that could help predict the surgical difficulty of LC after PTGBD in patients with AC. Figure 7 intuitively shows the ranking of these indicators, with each point representing a particular feature. A positive point value indicates a positive impact on output. The darker the color of the point, the greater the influence of the feature variable on the target result, and vice versa. The graph shows that the higher the corresponding values of Neut%, time until surgery after PTGBD, interval between disease onset and PTGBD, WBC count, history of upper abdominal surgery, gallstones, and GBWT, the more difficult it was to perform LC after PTGBD (Fig. 7). Based on the SHAP summary plot, seven influential SHAP dependency plots were derived to elucidate the role of these clinical indicators in predicting the surgical difficulty of LC after PTGBD (Fig. 8). In the SHAP dependency plots, the vertical axis represents the SHAP value of the clinical features, whereas the horizontal axis represents the range of variation in the clinical features, where a SHAP value above zero indicates greater difficulty in performing LC after PTGBD.

### Establishment of an online web calculator

To facilitate clinical application, we developed an online web calculator based on the NB model to predict the surgical difficulty of LC after PTGBD. The online web calculator (https://zw17786325639.shinyapps.io/difficulty/) allows users to input the patients’ corresponding clinical indicators and estimate the surgical difficulty of LC. In clinical practice, we can input patient data such as the interval between onset and PTGBD, history of upper abdominal surgery, WBC count, presence of gallstones, GBWT, Neut%, and time until surgery after PTGBD into a web calculator. Subsequently, the application integrates these factors and automatically calculates the difficulty of LC surgery after PTGBD.

## Discussion

AC is a highly prevalent acute abdominal condition associated with significant mortality. Its incidence has gradually increased with the aging global population [14]. It has atypical clinical symptoms, rapid progression, and diverse complications that significantly increase the complexity and risk of treatment. PTGBD offers the advantages of quickly relieving symptoms and avoiding the risk of emergency surgery. Furthermore, it provides a sufficient time window for subsequent LC, enabling patients to undergo surgery in the best possible state, thereby improving the safety and success rate of surgery [15]. However, it is important to recognize that PTGBD does not achieve a curative effect in AC; it serves only as a temporary measure for symptom control and stabilization. Consequently, the optimal timing for definitive surgery must be carefully selected based on the clinical status [16].

Particularly in older patients who often tolerate surgery poorly owing to decreased physiological function, thereby increasing the risk during LC, the challenges associated with PTGBD-followed LC become more pronounced [17]. Studies have shown that compared with primary LC without prior drainage, LC surgery after PTGBD is associated with a significantly longer average operative time, increased intraoperative blood loss, higher conversion rates to open laparotomy, and elevated postoperative complication rates [18]. These findings suggest that although PTGBD can alleviate AC symptoms in patients in a timely manner, it can also increase the difficulty and risk of subsequent LC. This may be related to the fact that PTGBD does not completely resolve edema and adhesions of the gallbladder and surrounding tissues [19]. Therefore, a detailed preoperative evaluation and preparation, adequate optimization of surgical strategies, and ensuring that patients undergo surgery in the best conditions are key to improving the success rate of LC after PTGBD in patients with AC.

In this study, we paid special attention to the rigor of the research methods, strictly screening cases according to the inclusion and exclusion criteria to ensure that our research participants had high homogeneity in the outcomes of LC, thus avoiding bias in the research results due to case differences. Our study’s novelty lies in the precise reverse analysis of clinical data from the DLC and NDLC groups, aimed at identifying key factors that may lead to difficult LC, thereby providing more personalized advice for clinicians, helping doctors better predict and judge the difficulty level of LC surgery, determining the best timing for surgery, and achieving the best surgical treatment outcomes. Our analysis revealed that the interval between onset and PTGBD, history of upper abdominal surgery, WBC count, presence of gallstones, GBWT, Neut%, and time until surgery after PTGBD were independent predictive factors for the difficulty of LC surgery in patients with AC after PTGBD. Based on these factors, we developed and verified eight machine-learning models to predict the difficulty of LCs in patients with AC after PTGBD. We found that the NB model had good predictive performance and precision in the training set (AUROC, 0.881; AUPRC, 0.886), which was also confirmed in the validation set (AUROC, 0.868; AUPRC, 0.858). Moreover, the calibration curve of the NB model was highly consistent with the fitting line, reflecting the high accuracy of the prediction model. The DCA further demonstrated the strong clinical applicability of the NB model. Therefore, a comprehensive evaluation indicated that the NB model was optimal. We utilized the SHAP analysis to create intuitive visualizations that illustrate the relative importance of each input variable. Ultimately, seven clinical features (Neut%, time until surgery after PTGBD, interval between symptom onset and PTGBD, WBC count, history of upper abdominal surgery, gallstones, and GBWT) were identified as key risk factors that distinguished DLC from NDLC. Based on these findings, we developed a dynamic prediction program and established an online calculator to predict the difficulty of LC in patients with AC after PTGBD, thereby enhancing the applicability of the model.

Research findings indicate that performing PTGBD within two days after symptom onset can more effectively reduce adhesions in the gallbladder and surrounding tissues, thereby creating more favorable conditions for subsequent LC [20]. A retrospective cohort study conducted by Bingener-Casey et al. showed that patients with a history of abdominal surgery had a significantly increased risk of complications during LC, which was approximately 30% higher than that in patients without such a history. A history of abdominal surgery, particularly of the upper abdomen, significantly increases the difficulty of LC [21]. Our study results showed that LC was significantly more difficult in patients with elevated WBC counts, probably because the inflammatory response led to congestion, edema, and adhesions in the gallbladder and surrounding tissues, thereby increasing the complexity and risk of surgery [22, 23]. Thicker gallbladder walls usually indicate more severe inflammation and fibrosis, which can increase adhesions between the gallbladder and surrounding tissues, making the anatomical structure more complex and increasing the difficulty and risk of surgery [24]. The presence of gallstones is another important factor in the complexity of LC; stones can not only cause obstruction of the cystic duct, triggering acute cholecystitis, but may also increase the risk of bile duct injury [25]. Studies have shown that if the interval between PTGBD and LC is too short, inflammation and adhesions of the gallbladder and surrounding tissues may not be completely resolved, thereby increasing the difficulty and risk of surgical procedures [26]. Excessive intervals can lead to gallbladder atrophy and fibrous adhesions of the surrounding tissues, which also increase the complexity of surgery [27]. Therefore, choosing the appropriate interval between surgeries is the key to improving the success rate of surgery.

Assessing the risk factors for surgical difficulty and constructing a predictive scoring system for LC in patients with AC after PTGBD are highly complex and pose a significant challenge for experienced clinicians. Machine learning approaches offer a superior solution to this problem by providing new methods and insights for creating clinically applicable predictive models and revolutionizing surgical difficulty assessment strategies. In this study, we employed the optimal NB algorithm and combined it with the SHAP analysis to confirm and quantify the impact direction and relative importance of each predictive variable on the risk of difficult LC after PTGBD. SHAP values indicated a higher Neut%, longer time until surgery after PTGBD, longer intervals between symptom onset and PTGBD, higher WBC count, thicker GBWT, the presence of gallstones, and a history of upper abdominal surgery, consistently pushed the model prediction towards higher surgical difficulty (positive SHAP values). Notably, in our model, Neut% had the highest predictive contribution, followed by the two time-interval variables. This underscores the critical role of the ongoing inflammatory response and the timing of surgery in predicting surgical difficulty. These findings align with those of previous studies, which suggest that a short interval between PTGBD and LC may increase difficulty due to unresolved acute inflammation and adhesions, whereas an interval that is too long may lead to gallbladder atrophy and fibrous adhesions of surrounding tissues, both of which increase surgical complexity [26, 27]. It is important to emphasize that, although the SHAP analysis effectively revealed the impact direction and relative strength of each variable within the model, it did not define precise clinical decision thresholds. To compensate for this and provide a more quantitative description of risk changes, we can combine SHAP analysis with multivariate logistic regression analysis to offer quantitative estimates of risk increase. For example, each additional day of delay in performing LC after PTGBD increases the likelihood of surgical difficulty by 3% (OR = 1.03, 95% CI: 1.02–1.05), while a history of upper abdominal surgery more than doubles the risk of difficult surgery (OR = 2.52, 95% CI: 1.59–4.03). These ORs, combined with the SHAP visualizations, reinforced the significant association between these seven predictive factors and the difficulty of LC after PTGBD.

The ultimate goal of constructing this model was to enhance the convenience of clinical applications and assist in clinical decision-making. To this end, based on the NB algorithm, we have developed an accessible online calculator website (https://zw17786325639.shinyapps.io/difficulty/). This tool enables clinicians to quickly and individually assess the surgical difficulty of LC after PTGBD in patients with AC, thereby providing support for selecting the appropriate timing of surgery and creating relatively safe surgical conditions, ultimately ensuring the safety of the surgery and reducing the incidence of postoperative complications.

However, some limitations must be continuously optimized and improved in clinical practice. First, because this was a retrospective study, it inevitably has a potential bias. For example, differences in technical skills among different centers or surgeons may introduce confounding factors. Second, some preoperative variables may have been omitted and were not included in the study, which may have led to the absence of important predictive factors for LC difficulty after PTGBD in patients with AC. Therefore, future studies should use external data from other institutions to validate the reproducibility of our findings. Further prospective studies and large-scale external validations incorporating a broader range of preoperative variables are crucial for improving the model and promoting its clinical application.

## Conclusion

In summary, we constructed a predictive model with good performance based on independent predictors (interval between disease onset and PTGBD, history of upper abdominal surgery, WBC count, gallstones, GBWT, Neut%, and time until surgery). PTGBD) can predict LC difficulty after PTGBD in patients with AC. Additionally, we developed an online calculator to translate our research results into clinical practice. This personalized prediction tool is helpful for assessing the surgical difficulty of LC after PTGBD in patients with AC and is of great significance for decision-making and management regarding the optimal timing of LC.

## Data Availability

The datasets generated and/or analyzed in the current study can be obtained from the corresponding author upon reasonable request.

## References

[1] Deng B et al (2024) Comparison of the analgesic effect of Dezocine and Esketamine in combination with sufentanil respectively after laparoscopic cholecystectomy: a prospective randomized controlled study. BMC Anesthesiol 24(1):51 38317099 10.1186/s12871-024-02430-y PMC10840296

[2] Mori Y et al (2018) Tokyo guidelines 2018: management strategies for gallbladder drainage in patients with acute cholecystitis (with videos). J Hepatobiliary Pancreat Sci 25(1):87–95 28888080 10.1002/jhbp.504

[3] Inoue K et al (2017) Optimal timing of cholecystectomy after percutaneous gallbladder drainage for severe cholecystitis. BMC Gastroenterol 17(1):71 28569137 10.1186/s12876-017-0631-8 PMC5452332

[4] Altieri MS et al (2020) Early cholecystectomy (≤ 8 weeks) following percutaneous cholecystostomy tube placement is associated with higher morbidity. Surg Endosc 34(7):3057–3063 31372890 10.1007/s00464-019-07050-z

[5] Lyu Y, Wang B (2024) Predictors of the difficulty of laparoscopic cholecystectomy after percutaneous transhepatic gallbladder drainage for grade II acute cholecystitis, vol 34. Surg Laparosc Endosc Percutan Tech, pp 479–484. 5 10.1097/SLE.0000000000001304 PMC11446531 39016308

[6] Di Buono G et al (2021) Difficult laparoscopic cholecystectomy and preoperative predictive factors. Sci Rep 11(1):2559 33510220 10.1038/s41598-021-81938-6 PMC7844234

[7] Bourgouin S et al (2016) How to predict difficult laparoscopic cholecystectomy? Proposal for a simple preoperative scoring system. Am J Surg 212(5):873–881 27329073 10.1016/j.amjsurg.2016.04.003

[8] Takada T (2018) Tokyo guidelines 2018: updated Tokyo guidelines for the management of acute cholangitis/acute cholecystitis. J Hepatobiliary Pancreat Sci 25(1):1–2 29334699 10.1002/jhbp.526

[9] Yokoe M et al (2018) Tokyo guidelines 2018: diagnostic criteria and severity grading of acute cholecystitis (with videos). J Hepatobiliary Pancreat Sci 25(1):41–54 29032636 10.1002/jhbp.515

[10] Ashfaq A et al (2016) The difficult gall bladder: outcomes following laparoscopic cholecystectomy and the need for open conversion. Am J Surg 212(6):1261–1264 28340928 10.1016/j.amjsurg.2016.09.024

[11] Inoue K et al (2017) Risk factors for difficulty of laparoscopic cholecystectomy in grade II acute cholecystitis according to the Tokyo guidelines 2013. BMC Surg 17(1):114 29183352 10.1186/s12893-017-0319-6 PMC5706415

[12] Hayama S et al (2016) Risk factors for difficult laparoscopic cholecystectomy in acute cholecystitis. Jsls 20(4):216–00065 10.4293/JSLS.2016.00065 PMC5081400 27807397

[13] Matsumoto M et al (2022) New scoring system for prediction of surgical difficulty during laparoscopic cholecystectomy after percutaneous transhepatic gallbladder drainage. Ann Gastroenterol Surg 6(2):296–306 35261956 10.1002/ags3.12522 PMC8889863

[14] Yokoe M et al (2017) Descriptive review of acute cholecystitis: Japan-Taiwan collaborative epidemiological study. J Hepatobiliary Pancreat Sci 24(6):319–328 28316140 10.1002/jhbp.450

[15] Melin MM et al (1995) Percutaneous cholecystostomy: a valuable technique in high-risk patients with presumed acute cholecystitis. Br J Surg 82(9):1274–1277 7552017 10.1002/bjs.1800820939

[16] Woodward SG et al (2021) Finding the most favorable timing for cholecystectomy after percutaneous cholecystostomy tube placement: an analysis of institutional and National data. J Am Coll Surg 232(1):55–64 33098966 10.1016/j.jamcollsurg.2020.10.010

[17] Miyasaka Y, Nakamura M, Wakabayashi G (2018) Pioneers in laparoscopic hepato-biliary-pancreatic surgery. J Hepatobiliary Pancreat Sci 25(1):109–111 28963814 10.1002/jhbp.506

[18] Fujinaga A et al (2021) Efficacy of releasing impacted gallstones after percutaneous transhepatic gallbladder drainage for acute cholecystitis and consideration of the surgical difficulty during laparoscopic cholecystectomy. J Hepatobiliary Pancreat Sci 28(11):993–999 33128850 10.1002/jhbp.857

[19] Liu YQ et al (2023) Increased difficulty and complications of delayed laparoscopic cholecystectomy following percutaneous transhepatic gallbladder drainage in acute cholecystitis: a retrospective study. BMC Surg 23(1):277 37704959 10.1186/s12893-023-02185-2 PMC10500720

[20] Hadad SM et al (2007) Delay from symptom onset increases the conversion rate in laparoscopic cholecystectomy for acute cholecystitis. World J Surg 31(6):1298–1201. discussion 1302-3 17483986 10.1007/s00268-007-9050-2

[21] Bingener-Casey J et al (2002) Reasons for conversion from laparoscopic to open cholecystectomy: a 10-year review. J Gastrointest Surg 6(6):800–805 12504217 10.1016/s1091-255x(02)00064-1

[22] Sakuramoto S et al (2000) Preoperative evaluation to predict technical difficulties of laparoscopic cholecystectomy on the basis of histological inflammation findings on resected gallbladder. Am J Surg 179(2):114–121 10773146 10.1016/s0002-9610(00)00248-8

[23] Lipman JM et al (2007) Preoperative findings predict conversion from laparoscopic to open cholecystectomy. Surgery 142(4):556–563. discussion 563-5 17950348 10.1016/j.surg.2007.07.010

[24] Majeski J (2007) Significance of preoperative ultrasound measurement of gallbladder wall thickness. Am Surg 73(9):926–929 17939429

[25] Polychronidis A et al (2008) Laparoscopic cholecystectomy in elderly patients. J Gastrointestin Liver Dis 17(3):309–313 18836625

[26] Yamada K et al (2015) Optimal timing for performing percutaneous transhepatic gallbladder drainage and subsequent cholecystectomy for better management of acute cholecystitis. J Hepatobiliary Pancreat Sci 22(12):855–861 26479740 10.1002/jhbp.294

[27] Lo CM et al (1998) Prospective randomized study of early versus delayed laparoscopic cholecystectomy for acute cholecystitis. Ann Surg 227(4):461–467 9563529 10.1097/00000658-199804000-00001 PMC1191296

